# Association of Preoperative Pan-Immune-Inflammation Value with Adverse Pathology and Oncological Outcomes Following Radical Prostatectomy: A Single-Center Retrospective Study

**DOI:** 10.7150/ijms.139320

**Published:** 2026-09-03

**Authors:** Tsz-Yi Tang, Hao-Han Chang, Yung-Shun Juan, Yung-Chin Lee, Chia-Yang Lee, Jiun-Hung Geng, Shu-Pin Huang

**Affiliations:** 1 Graduate Institute of Clinical Medicine, College of Medicine, Kaohsiung Medical University, Kaohsiung, Taiwan.; 2 Department of Urology, Kaohsiung Municipal Siaogang Hospital, Kaohsiung Medical University, Kaohsiung, Taiwan.; 3 Department of Urology, Kaohsiung Medical University Hospital, Kaohsiung Medical University, Kaohsiung, Taiwan.; 4 Department of Urology, School of Medicine, College of Medicine, Kaohsiung Medical University, Kaohsiung, Taiwan.

**Keywords:** prostate cancer, radical prostatectomy, pan-immune-inflammation value, systemic inflammation, androgen deprivation therapy, overall survival

## Abstract

**Background:**

High Pan-Immune-Inflammation Value (PIV) has been associated with poorer survival outcomes in men with metastatic prostate cancer. However, whether PIV is associated with adverse pathology and long-term oncological outcomes in localized prostate cancer treated with radical prostatectomy (RP) remains unclear.

**Methods:**

We retrospectively evaluated 808 men who underwent RP for prostate adenocarcinoma at Kaohsiung Medical University Hospital between 2012 and 2023; 442 met the eligibility criteria and were included in the final analysis. PIV was dichotomized using a Youden-derived cutpoint (153) based on subsequent androgen deprivation therapy (ADT), defined as any adjuvant or salvage ADT administered after RP. We used multivariable logistic and Cox regression models to evaluate associations between preoperative PIV and adverse pathological features, subsequent ADT, biochemical recurrence (BCR), metastasis-free survival (MFS), and overall survival (OS).

**Results:**

Median follow-up was 65.8 months. During follow-up, 100 men (22.6%) received subsequent ADT, 71 (16.1%) developed metastasis or died, and 54 (12.2%) died. High PIV was independently associated with ≥pT3 disease (adjusted odds ratio [aOR] 1.79, 95% confidence interval [CI] 1.14-2.81; p = 0.012), subsequent ADT (adjusted hazard ratio [aHR] 2.18, 95% CI 1.32-3.60; p = 0.002), shorter MFS (aHR 2.37, 95% CI 1.29-4.35; p = 0.006), and shorter OS (aHR 3.12, 95% CI 1.46-6.67; p = 0.003), but not with BCR.

**Conclusions:**

High preoperative PIV was independently associated with ≥pT3 disease, subsequent ADT, shorter MFS, and shorter OS after RP, but not with BCR. These findings suggest that PIV may reflect disease progression beyond PSA-defined recurrence and may serve as a complementary preoperative biomarker. Further validation in independent cohorts is required.

## Introduction

Prostate cancer is the second most commonly diagnosed malignancy in men worldwide, and its global burden continues to grow 1, 2. However, despite curative intent, approximately 20-50% of patients develop biochemical recurrence within 10 years after treatment, and a subset subsequently progresses to metastatic disease 3. Preoperative risk stratification still relies mainly on the serum prostate-specific antigen (PSA), biopsy Gleason grade group, clinical stage, and multiparametric magnetic resonance imaging 4, 5. However, predicting postoperative outcomes remains challenging, highlighting the need for simple and readily available biomarkers to improve preoperative risk assessment.

Systemic inflammation is increasingly recognized as a hallmark of cancer. It contributes to tumor progression, invasion, and metastatic dissemination 6, 7, and platelets interact directly with tumor cells to facilitate spread 8. The pan-immune-inflammation value (PIV), derived from the neutrophil, platelet, monocyte, and lymphocyte counts of a routine blood test, integrates these immune compartments into a single index. It was originally developed in metastatic colorectal cancer, where it demonstrated stronger prognostic performance than conventional inflammation-based biomarkers and was independently associated with survival outcomes 9. Similar associations have been reported in other malignancies, including breast cancer, lung cancer, and esophageal cancer, where elevated PIV has been linked to poorer treatment response and survival 10, 11. In prostate cancer, a higher PIV has been associated with shorter overall survival (OS) in metastatic castration-resistant disease 12 and with the presence of malignancy at biopsy in men with a PSA level of 4-20 ng/mL 13. Because PIV reflects the systemic inflammatory burden generated by a tumor, it may also mirror the aggressiveness of localized disease before surgery. Other blood-based inflammation indices also have been linked to adverse pathology, biochemical recurrence, and survival after RP 14-17, consistent with their prognostic value across solid tumors 18, 19.

Evidence regarding the association between preoperative PIV and postoperative treatment requirements and long-term oncological outcomes after curative surgery remains limited. This gap is clinically important because biochemical recurrence, defined by PSA alone, is heterogeneous and does not reliably predict metastasis or mortality 20, 21. In contrast, the need for postoperative systemic therapy may better reflect disease progression. Whether preoperative PIV can identify men who will require postoperative systemic therapy remains unknown.

Thus, we aimed to evaluate the association of preoperative PIV with adverse pathological features after radical prostatectomy and with postoperative outcomes, including subsequent androgen deprivation therapy (ADT), metastasis-free survival (MFS), OS, and biochemical recurrence (BCR). The lymphocyte-to-monocyte ratio (LMR) was reported as a biologically opposing comparator.

## Materials and Methods

### Study cohort

We retrospectively reviewed consecutive men who underwent radical prostatectomy (RP) for prostate cancer at Kaohsiung Medical University Hospital (KMUH) between 2012 and 2023. Of 811 patients identified, three with non-prostate cancer pathology were excluded, leaving 808 men for analysis. We obtained clinical and pathological characteristics, preoperative laboratory data, PSA measurements, and treatment details from electronic medical records and the institutional cancer registry. The study was conducted in accordance with the Declaration of Helsinki and approved by the Institutional Review Board of Kaohsiung Medical University Hospital (KMUHIRB-E(I)-20260221). Informed consent was not required because of the retrospective study design.

### Preoperative PIV assessment

A complete preoperative differential count was available in 483 men. We excluded 41 men with confounding counts (leukocytosis, > 11 × 10³/µL; leukopenia, < 4 × 10³/µL), leaving 442 men in the final analytical cohort (Figure 1). PIV was calculated as neutrophils × platelets × monocytes/ lymphocytes. We identified the preoperative PIV cutpoint (153) using the Youden index for subsequent ADT, and divided the population into low- and high-PIV groups. Preoperative PIV was right-skewed (median 196.7, IQR 131.4-323.1, range 23.8-2674.7; skewness 4.4); LMR (lymphocytes/monocytes) was summarized descriptively as a comparator. The distribution of PIV and LMR is shown in Supplementary Figure S1.

### Outcomes

Subsequent ADT was defined as any adjuvant or salvage ADT initiated after radical prostatectomy and was considered a treatment-decision outcome reflecting real-world postoperative management. Grade-group upgrading was defined as a higher pathological grade group in the radical prostatectomy specimen than at biopsy. BCR was defined as two consecutive PSA measurements ≥ 0.2 ng/mL following an initially undetectable postoperative PSA level. BCR was evaluable in 321 of the 442 men; the remaining 121 were excluded from BCR analyses because their postoperative PSA data were insufficient to determine BCR status (no documented undetectable postoperative PSA nadir, or fewer than two postoperative PSA measurements). MFS was defined as the time from radical prostatectomy to radiographic metastasis or death from any cause, whichever occurred first 22. OS was defined as the time from radical prostatectomy to death from any cause. We calculated time-to-event outcomes from the date of radical prostatectomy to the occurrence of the respective endpoint or last follow-up. For the subsequent ADT analysis, we measured time from the date of radical prostatectomy to the initiation of adjuvant or salvage ADT. We excluded 26 men because the postoperative interval to subsequent ADT could not be defined, leaving 416 men for analysis. Metastasis-free survival and overall survival were both measured from the date of radical prostatectomy and therefore included all 442 men.

### Statistical analysis

Descriptive analyses used absolute frequencies and percentages for categorical variables and medians with interquartile ranges (IQRs) for continuous variables. Categorical variables were compared using the χ² test or Fisher's exact test, as appropriate, and continuous variables using the Mann-Whitney U test. The preoperative PIV and LMR cutpoints were identified using the Youden index from receiver-operating characteristic (ROC) analysis for subsequent ADT. Associations between PIV and adverse pathological outcomes (≥pT3 disease, pathological grade group ≥ 4, lymph-node metastasis, grade-group upgrading, and positive surgical margin [PSM]) were evaluated using multivariable logistic regression models adjusted for age, diagnostic grade group, initial PSA (per ng/mL) and clinical T stage, with results reported as odds ratios (ORs) and 95% confidence intervals (CIs). We used the Kaplan-Meier method to estimate survival curves for subsequent ADT, MFS, and OS, and compared them using the log-rank test. Univariable and multivariable Cox proportional-hazards regression models were used to evaluate associations between PIV and time-to-event outcomes, reporting results as adjusted hazard ratios (aHRs) and 95% CIs. Covariates included in multivariable models were selected a priori based on established clinical relevance and previous prostate cancer prognostic models. Multivariable models for subsequent ADT, MFS, and OS were adjusted for the same pre-specified preoperative covariates: age, Charlson comorbidity index, log-transformed initial PSA, clinical T stage, and diagnostic grade group. Continuous covariates were modeled per unit, whereas PIV was analyzed as a dichotomous variable using the Youden-derived cutpoint. The proportional-hazards assumption was assessed using Schoenfeld residuals; no violation was detected (global p = 0.17, 0.31, and 0.11 for subsequent ADT, MFS, and OS, respectively). Because PIV was primarily analyzed using a data-derived dichotomous cutpoint, we further explored the association between PIV and the risk of subsequent ADT using a univariable restricted cubic spline model to assess the shape and potential non-linearity of the dose-response relationship. The spline model used three knots placed at the 10th, 50th, and 90th percentiles of the log-transformed PIV distribution and was truncated at the 95th percentile. In addition, we performed sensitivity analyses using PIV tertiles to evaluate whether the observed associations were robust to alternative PIV categorizations. All statistical analyses were conducted using SAS version 9.4 and R version 4.5.2. Statistical significance was defined as a two-sided p-value of < 0.05. The authors used Claude (Anthropic) to assist with language editing and structural revision of the manuscript.

## Results

### Patients

Among 811 men who underwent radical prostatectomy, 808 had prostate adenocarcinoma, and preoperative differential blood counts were available for 483. After excluding 17 men with leukocytosis and 24 with leukopenia, 442 remained in the analytical cohort: 148 with low PIV and 294 with high PIV (Figure 1). The median follow-up was 65.8 months.

Table 1 shows baseline characteristics. Men with high PIV were more likely to have ≥ pT3 disease (51.7% vs 37.2%, p = 0.004) and to receive subsequent ADT (27.2% vs 13.5%, p = 0.001). They also experienced more MFS events, defined as metastasis or death from any cause (19.7% vs 8.8%, p = 0.003), and higher all-cause mortality (15.6% vs 5.4%, p = 0.002). As expected, LMR was lower in the high-PIV group (median 3.5 vs 5.5, p < 0.001). Overall, 100 men (22.6%) received subsequent ADT and 54 (12.2%) died. Postoperative radiotherapy was administered to 70 men (15.8%) and did not differ between the high- and low-PIV groups (14.6% vs 18.2%, p = 0.326).

### PIV and adverse pathology

Supplementary Table S1 shows associations between preoperative PIV and adverse pathological outcomes. In multivariable logistic regression, high PIV was independently associated with ≥ pT3 disease (aOR 1.79, 95% CI 1.14-2.81, p = 0.012). No significant associations were observed for pathological grade group ≥ 4 disease (OR 1.29, 95% CI 0.79-2.13, p = 0.309), lymph-node metastasis (OR 0.87, 95% CI 0.35-2.16, p = 0.762), grade-group upgrading (OR 1.40, 95% CI 0.90-2.17, p = 0.136), or PSM (OR 0.99, 95% CI 0.66-1.49, p = 0.968).

### PIV and clinical outcomes after RP

Men with high PIV were more likely to receive subsequent ADT than those with low PIV (log-rank p = 0.001; Figure 2A). In Cox regression analyses, high PIV was associated with a shorter time to subsequent ADT in both univariable (HR 2.22, 95% CI 1.36-3.63, p = 0.001) and multivariable models (aHR 2.18, 95% CI 1.32-3.60, p = 0.002; Table 2). MFS was also shorter in the high-PIV group (log-rank p = 0.003; Figure 2B), and the association persisted after adjustment (aHR 2.37, 95% CI 1.29-4.35, p = 0.006; Table 2). OS was also shorter (log-rank p = 0.002; Figure 2C), and high PIV was independently associated with all-cause mortality (aHR 3.12, 95% CI 1.46-6.67, p = 0.003; Table 2). In contrast, preoperative PIV was not associated with BCR (aHR 0.99, 95% CI 0.62-1.60, p = 0.98; n = 321).

### Spline and sensitivity analyses

The univariable (unadjusted) restricted cubic spline analysis demonstrated a positive association between preoperative PIV and the risk of subsequent ADT (overall p = 0.017), with no evidence of non-linearity (non-linear p = 0.169; Supplementary Figure S2). When PIV was categorized into tertiles, the risks of subsequent ADT, metastasis, or death increased progressively across tertiles (univariable Cox linear-trend p = 0.005, 0.003, and < 0.001, respectively; Supplementary Figure S3), supporting the robustness of the primary findings.

## Discussion

In this single-center cohort of men undergoing radical prostatectomy for clinically localized prostate cancer, a higher preoperative PIV was independently associated with ≥ pT3 disease, subsequent ADT, shorter MFS, and shorter OS, but not with BCR. Patients with a high PIV had approximately 2-fold higher risks of subsequent ADT and of metastasis or death and a more than 3-fold higher risk of death compared with those with a low PIV.

Interestingly, there was no connection between preoperative PIV and BCR. BCR is defined solely by a rise in prostate-specific antigen (PSA) and represents a heterogeneous event that does not necessarily translate into metastatic progression or mortality. Consequently, BCR is only modestly associated with long-term survival outcomes and remains an imperfect surrogate for disease progression 3, 20. Even persistent postoperative PSA captures only part of the prognostic picture 23, 24. By contrast, MFS has been formally validated as a strong surrogate of OS in localized prostate cancer in a meta-analysis of nearly 29,000 men 22, and subsequent ADT generally reflects clinically significant disease progression. The differential association of PIV with these outcomes suggests that the systemic inflammatory milieu reflected by PIV may be more closely linked to tumor aggressiveness and metastatic potential than to PSA kinetics alone 25-28. Although the initiation of subsequent ADT is a clinical decision rather than a purely biological event, we consider this property informative in the real-world setting: subsequent ADT captures the actual treatment burden experienced by patients and integrates guideline-driven triggers with individualized judgment that takes age, comorbidity, and patient preference into account, and therefore reflects how disease progression translates into management in routine practice. In this sense, the treatment-decision outcome complements the objective endpoints of metastasis and death analyzed in parallel.

Our findings build on a growing body of evidence supporting the prognostic value of systemic inflammatory biomarkers in prostate cancer. The neutrophil-to-lymphocyte ratio, platelet-to-lymphocyte ratio, and systemic immune-inflammation index have each been associated with adverse pathology and poorer oncological outcomes across the diagnostic, post-prostatectomy, and salvage settings 14-16, 29, 30. Evidence specific to PIV in prostate cancer, however, comes largely from two settings: predicting cancer at diagnostic biopsy 13, 31, 32 and Lutetium-177-PSMA-617 radioligand therapy, in which HRs for OS of approximately 2.9 to 4.3 have been reported 33-35. In the present study, the aHR for OS was 3.12, comparable to estimates reported in metastatic prostate cancer cohorts. This finding is also consistent with a recent meta-analysis of nearly 9,000 patients across multiple tumor types, which demonstrated a significant association between elevated PIV and OS 11. Similar prognostic associations have been reported in lung 27, 36, breast 37, and gastric cancer 25. Taken together, these observations suggest that PIV may reflect a systemic inflammatory state associated with aggressive tumor biology across different disease stages and tumor types. Our study extends these findings to localized prostate cancer treated with radical prostatectomy.

Each component of PIV has a plausible role in tumor progression: neutrophils, platelets and monocytes facilitate tumor-cell survival, angiogenesis, immune evasion and the establishment of metastatic niches 6-8, whereas lymphocytes index the host adaptive anti-tumor response. By integrating these opposing immune compartments, PIV may provide a more comprehensive measure of the host inflammatory response than individual blood-cell ratios alone 9. PIV summarizes the balance between pro-tumoral inflammation and anti-tumoral immunity in a single number, which may explain why it has outperformed simpler ratios in several series and why a low PIV has been associated with greater pathological response to therapy 38.

PIV has several potential clinical advantages. It is inexpensive, widely available, and can be calculated from a routine preoperative blood test. Although PIV is unlikely to replace established prognostic factors, its independent association with subsequent ADT, MFS, and OS suggests that it may serve as a useful adjunct to existing preoperative risk-stratification tools. Further external validation is required before routine clinical implementation. Integration of PIV into multivariable nomograms, as has been done in other malignancies 39, and serial monitoring of PIV trajectories before and after treatment 40 are next steps that may sharpen its clinical utility. To our knowledge, this is the first study to evaluate the association between preoperative PIV and adverse pathological features, subsequent ADT, MFS, and OS following radical prostatectomy.

Several limitations warrant consideration. First, this retrospective study was restricted to men with an evaluable preoperative CBC-DC. Because testing was not performed at random, selection bias cannot be excluded. The direction and magnitude of any residual bias remain uncertain, and the findings may be most applicable to patients for whom a preoperative differential count is obtained. Second, the PIV cutpoint of 153 was derived and evaluated in the same cohort, potentially resulting in optimism and inflated dichotomized effect estimates. Although the tertile and spline analyses showed broadly consistent risk gradients, they do not validate 153 as a transferable threshold. This cutpoint should therefore be considered exploratory and may not be generalizable to other settings. Finally, the limited numbers of metastatic events and deaths reduced the precision of the MFS and OS estimates. External validation of both the observed associations and any proposed clinical cutpoint is required.

## Conclusion

Preoperative pan-immune-inflammation value (PIV) was independently associated with ≥ pT3 disease, subsequent androgen deprivation therapy, metastasis or death, and death after radical prostatectomy, but not with PSA-defined biochemical recurrence. These findings suggest that PIV captures clinically meaningful disease progression beyond biochemical recurrence.

## Supplementary Material

Supplementary figures.

## Figures and Tables

**Figure 1:**
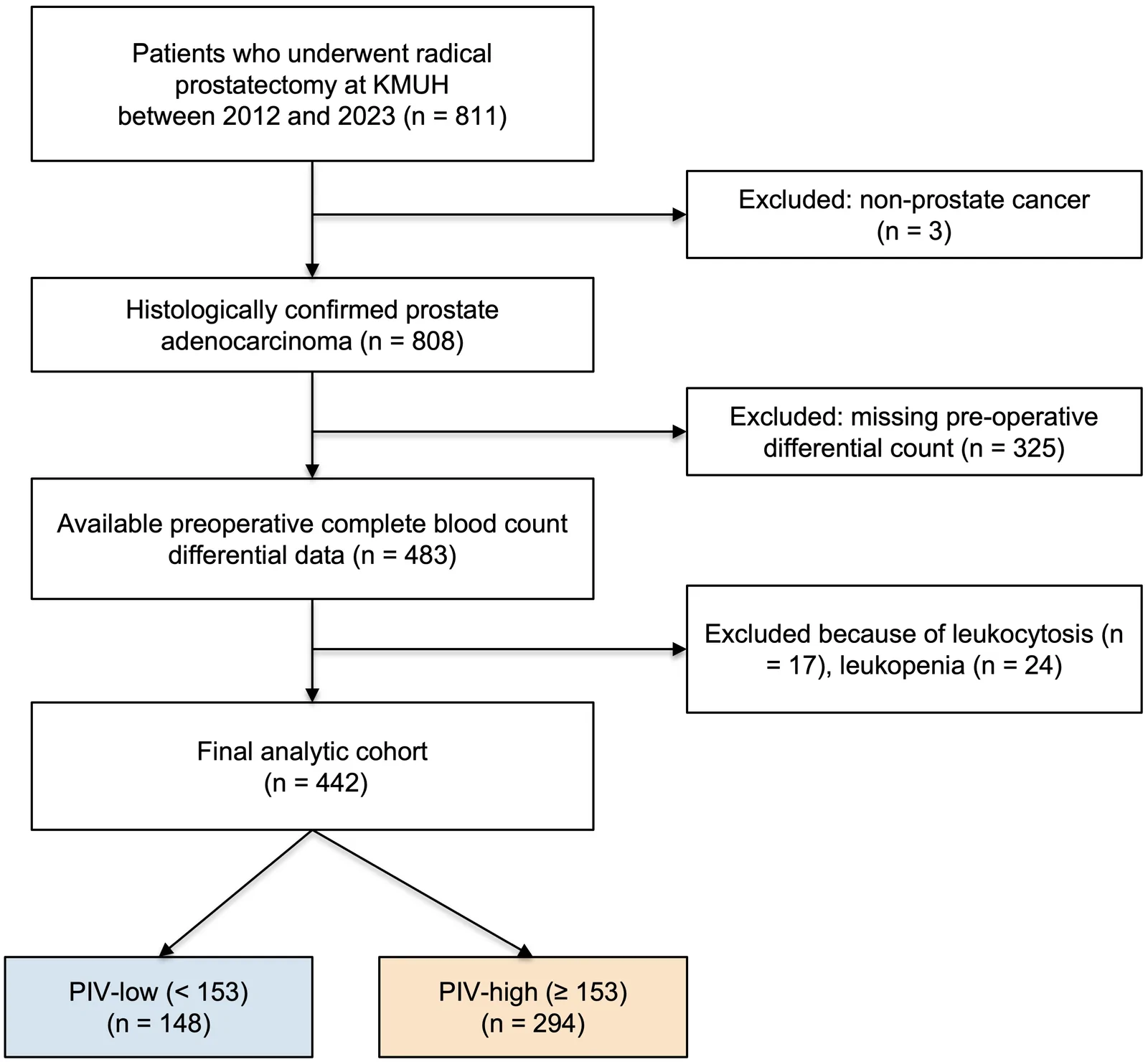
Study flow diagram of patient selection and stratification into low- and high-PIV groups.

**Figure 2:**
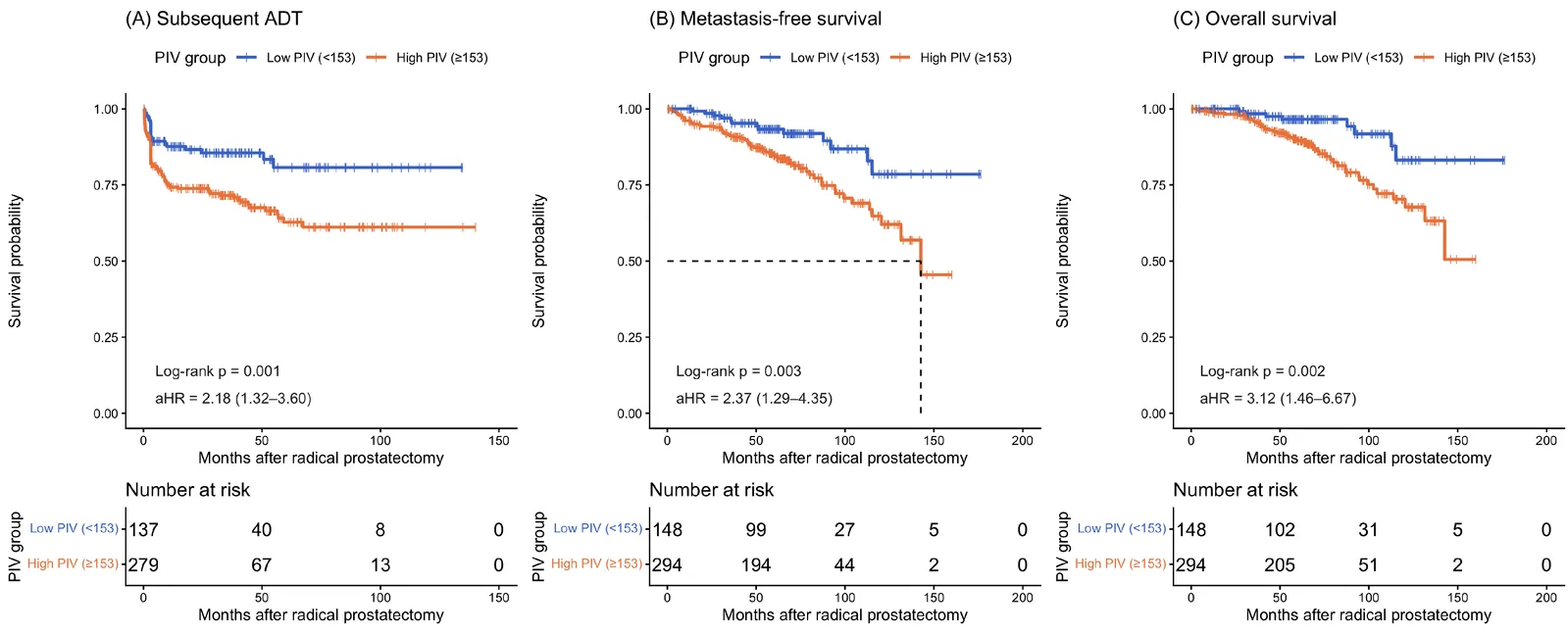
Kaplan-Meier curves for (A) subsequent androgen deprivation therapy (ADT), (B) metastasis-free survival (MFS), and (C) overall survival (OS) according to preoperative pan-immune-inflammation value (PIV) group. Log-rank p values and adjusted hazard ratios (aHRs) are displayed in each panel. The ADT analysis included 416 men because a valid postoperative interval to subsequent ADT could not be defined in 26 patients.

**Table 1:** Baseline clinicopathological characteristics stratified by PIV status in patients who underwent radical prostatectomy.

Characteristic	Total (n = 442)	Low PIV (n = 148)	High PIV (n = 294)	p
Age, years	69.0 (64.9-72.8)	68.8 (64.8-72.1)	69.0 (65.1-73.2)	0.364
Charlson comorbidity index				0.218
0	142 (32.1)	41 (27.7)	101 (34.4)	
1-2	182 (41.2)	69 (46.6)	113 (38.4)	
≥ 3	118 (26.7)	38 (25.7)	80 (27.2)	
Hypertension	257 (58.1)	82 (55.4)	175 (59.5)	0.407
Diabetes mellitus	114 (25.8)	32 (21.6)	82 (27.9)	0.155
Coronary artery disease	74 (16.7)	24 (16.2)	50 (17.0)	0.834
ECOG performance status				0.155
0	49 (11.1)	21 (14.2)	28 (9.5)	
1	373 (84.4)	116 (78.4)	257 (87.4)	
≥ 2	1 (0.2)	0 (0.0)	1 (0.3)	
Unknown	19 (4.3)	11 (7.4)	8 (2.7)	
Initial PSA, ng/mL	11.8 (7.2-23.2)	11.4 (7.0-20.4)	12.1 (7.3-25.5)	0.417
Clinical T stage				0.344
cT1	34 (7.7)	14 (9.5)	20 (6.8)	
cT2	319 (72.2)	109 (73.6)	210 (71.4)	
cT3-4	89 (20.1)	25 (16.9)	64 (21.8)	
Diagnostic grade group				0.184
1	163 (36.9)	57 (38.5)	106 (36.1)	
2	62 (14.0)	26 (17.6)	36 (12.2)	
3	82 (18.6)	30 (20.3)	52 (17.7)	
4	68 (15.4)	19 (12.8)	49 (16.7)	
5	67 (15.2)	16 (10.8)	51 (17.3)	
Pathological ISUP grade group				0.121
1	81 (18.3)	30 (20.3)	51 (17.3)	
2	100 (22.6)	40 (27.0)	60 (20.4)	
3	92 (20.8)	29 (19.6)	63 (21.4)	
4	61 (13.8)	23 (15.5)	38 (12.9)	
5	108 (24.4)	26 (17.6)	82 (27.9)	
Grade-group upgrading	190 (43.0)	60 (40.5)	130 (44.2)	0.424
Pathological stage ≥pT3	207 (46.8)	55 (37.2)	152 (51.7)	0.004
Lymph-node metastasis (pN1)	25 (7.8)	8 (7.5)	17 (7.9)	0.919
Not performed	120 (27.1)	42 (28.4)	78 (26.5)	
PSM	200 (45.2)	65 (43.9)	135 (45.9)	0.690
PIV	196.7 (131.4-323.1)	110.0 (87.2-131.4)	264.2 (196.9-410.5)	< 0.001
LMR	4.1 (3.1-5.3)	5.5 (4.5-6.6)	3.5 (2.8-4.4)	< 0.001
Outcomes				
Subsequent ADT	100 (22.6)	20 (13.5)	80 (27.2)	0.001
Metastasis or death (MFS event)	71 (16.1)	13 (8.8)	58 (19.7)	0.003
Death (OS event)	54 (12.2)	8 (5.4)	46 (15.6)	0.002
Postoperative radiotherapy	70 (15.8)	27 (18.2)	43 (14.6)	0.326
BCR	79 (24.6)	26 (23.4)	53 (25.2)	0.720

Data are median (interquartile range) for continuous variables and n (%) for categorical variables. PIV was divided at 153 (Youden index for subsequent ADT). The p value compares low versus high PIV (Mann-Whitney U, or χ² / Fisher exact test). BCR is presented among the 321 men in whom BCR status was evaluable (low PIV, n = 111; high PIV, n = 210). IQR: interquartile range; ECOG: Eastern Cooperative Oncology Group; PSA: prostate-specific antigen; cT: clinical tumor stage; ISUP: International Society of Urological Pathology; pT: pathological tumor stage; pN: pathological lymph-node stage; PSM: positive surgical margin; PIV: pan-immune-inflammation value; LMR: lymphocyte-to-monocyte ratio; ADT: androgen deprivation therapy; MFS: metastasis-free survival; OS: overall survival; BCR: biochemical recurrence.

**Table 2:** Univariable and multivariable Cox regression models for subsequent androgen deprivation therapy, metastasis-free survival, and overall survival after radical prostatectomy.

Characteristic	Subsequent ADT				MFS				OS			
	Univariable		Multivariable		Univariable		Multivariable		Univariable		Multivariable	
	HR (95% CI)	p	HR (95% CI)	p	HR (95% CI)	p	HR (95% CI)	p	HR (95% CI)	p	HR (95% CI)	p
PIV, high vs low (≥ 153 vs < 153)	2.22 (1.36-3.63)	0.001	2.18 (1.32-3.60)	0.002	2.40 (1.32-4.39)	0.004	2.37 (1.29-4.35)	0.006	3.05 (1.43-6.46)	0.004	3.12 (1.46-6.67)	0.003
Age, per year	1.03 (1.00-1.07)	0.037	1.02 (0.98-1.05)	0.339	1.04 (1.00-1.08)	0.041	1.03 (0.99-1.07)	0.099	1.04 (1.00-1.09)	0.074	1.04 (0.99-1.09)	0.101
Charlson comorbidity index, per point	0.93 (0.83-1.04)	0.190	0.94 (0.84-1.05)	0.264	1.08 (0.97-1.19)	0.156	1.12 (1.01-1.25)	0.039	1.10 (0.98-1.23)	0.111	1.14 (1.01-1.29)	0.038
Initial PSA, per log ng/mL	1.72 (1.47-2.01)	< 0.001	1.56 (1.28-1.89)	< 0.001	1.24 (0.99-1.57)	0.065	1.26 (0.98-1.61)	0.073	1.04 (0.81-1.33)	0.777	1.07 (0.81-1.41)	0.621
Clinical T stage, cT3-4 vs cT1-2	2.23 (1.48-3.36)	< 0.001	1.40 (0.91-2.18)	0.129	1.23 (0.72-2.09)	0.441	1.06 (0.60-1.85)	0.845	0.77 (0.40-1.50)	0.444	0.66 (0.33-1.34)	0.252
Diagnostic grade group, per unit	1.49 (1.30-1.70)	< 0.001	1.29 (1.11-1.49)	< 0.001	1.08 (0.93-1.26)	0.296	0.98 (0.83-1.16)	0.802	1.07 (0.90-1.27)	0.467	1.03 (0.85-1.25)	0.757

Each multivariable model included all listed covariates simultaneously. Continuous covariates (age, Charlson comorbidity index, log-transformed initial PSA, diagnostic grade group) are expressed per unit; clinical T stage (cT3-4 vs cT1-2) and PIV (≥153 vs <153) are dichotomized. ADT: androgen deprivation therapy; CI: confidence interval; HR: hazard ratio; MFS: metastasis-free survival; OS: overall survival; PIV: pan-immune-inflammation value; PSA: prostate-specific antigen.

## Data Availability

The data that support the findings of this study are available from the corresponding author upon reasonable request. The data are not publicly available because they contain information that could compromise the privacy of research participants.

## Abbreviations

ADT: androgen deprivation therapy
aHR: adjusted hazard ratio
aOR: adjusted odds ratio
BCR: biochemical recurrence
CBC: complete blood count
CBC-DC: complete blood count with differential count
CI: confidence interval
HR: hazard ratio
ISUP: International Society of Urological Pathology
LMR: lymphocyte-to-monocyte ratio
MFS: metastasis-free survival
OR: odds ratio
OS: overall survival
PIV: pan-immune-inflammation value
PSA: prostate-specific antigen
PSM: positive surgical margin
ROC: receiver-operating-characteristic
RP: radical prostatectomy
