# Supplementary material for: Association of Preoperative Pan-Immune-Inflammation Value with Adverse Pathology and Oncological Outcomes Following Radical Prostatectomy: A Single-Center Retrospective Study
Source: Int J Med Sci. 2026 Sep 3;23(10):3220–7. doi: 10.7150/ijms.139320 (PMC13617502; doi:10.7150/ijms.139320)

Supplementary Materials

Supplementary Table S1. Multivariable logistic regression models evaluating the association between preoperative PIV and adverse pathological outcomes after radical prostatectomy.

	≥ pT3		Grade group ≥ 4		pN ≥ 1		Upgrading		PSM	
	**OR (95% CI)**	***p***	**OR (95% CI)**	***p***	**OR (95% CI)**	***p***	**OR (95% CI)**	***p***	**OR (95% CI)**	***p***
PIV (high vs low)	1.79 (1.14-2.81)	0.012	1.29 (0.79-2.13)	0.309	0.87 (0.35-2.16)	0.762	1.40 (0.90-2.17)	0.136	0.99 (0.66-1.49)	0.968
Age (per year)	1.01 (0.98-1.04)	0.581	1.03 (0.99-1.07)	0.142	1.06 (0.98-1.14)	0.144	1.01 (0.98-1.04)	0.557	1.01 (0.98-1.04)	0.418
Diagnostic grade group (per unit)	1.34 (1.15-1.56)	<0.001	2.19 (1.84-2.61)	<0.001	1.57 (1.14-2.16)	0.005	0.53 (0.45-0.62)	<0.001	1.23 (1.07-1.41)	0.003
Initial PSA, per ng/mL	1.04 (1.02-1.05)	<0.001	1.02 (1.01-1.03)	<0.001	1.00 (1.00-1.00)	0.793	1.00 (1.00-1.00)	0.604	1.00 (1.00-1.00)	0.934
Clinical T stage										
cT1-2	1.00		1.00		1.00		1.00		1.00	
cT3-4	2.00 (1.15-3.48)	0.014	0.69 (0.38-1.26)	0.230	1.14 (0.45-2.88)	0.775	1.55 (0.90-2.68)	0.114	1.76 (1.07-2.88)	0.025

Odds ratios (OR) with 95% CIs and p values are from one multivariable logistic model per outcome, each including PIV, age, diagnostic grade group, initial PSA (per ng/mL), and clinical T stage; all estimates are therefore adjusted for the other listed covariates. High and low PIV were defined as ≥153 and <153, respectively. CI: confidence interval; OR: odds ratio; PSM: positive surgical margin.

Supplementary figure

Supplementary Figure S1. Distribution of preoperative pan-immune-inflammation value (PIV) and lymphocyte-to-monocyte ratio (LMR) in the cohort (n = 442). (A) Preoperative PIV: right-skewed (median 197, IQR 131-323, range 24-2675, skewness 4.4); the orange dashed line marks the Youden-derived cutpoint at 153. (B) Preoperative LMR: median 4.10 (IQR 3.09-5.34, range 0.63-17.21, skewness 1.6); the orange dashed line marks the corresponding Youden-derived LMR value (3.0). LMR is shown for descriptive comparison only; PIV was the marker dichotomized at its Youden-derived cutpoint for the primary survival analyses.

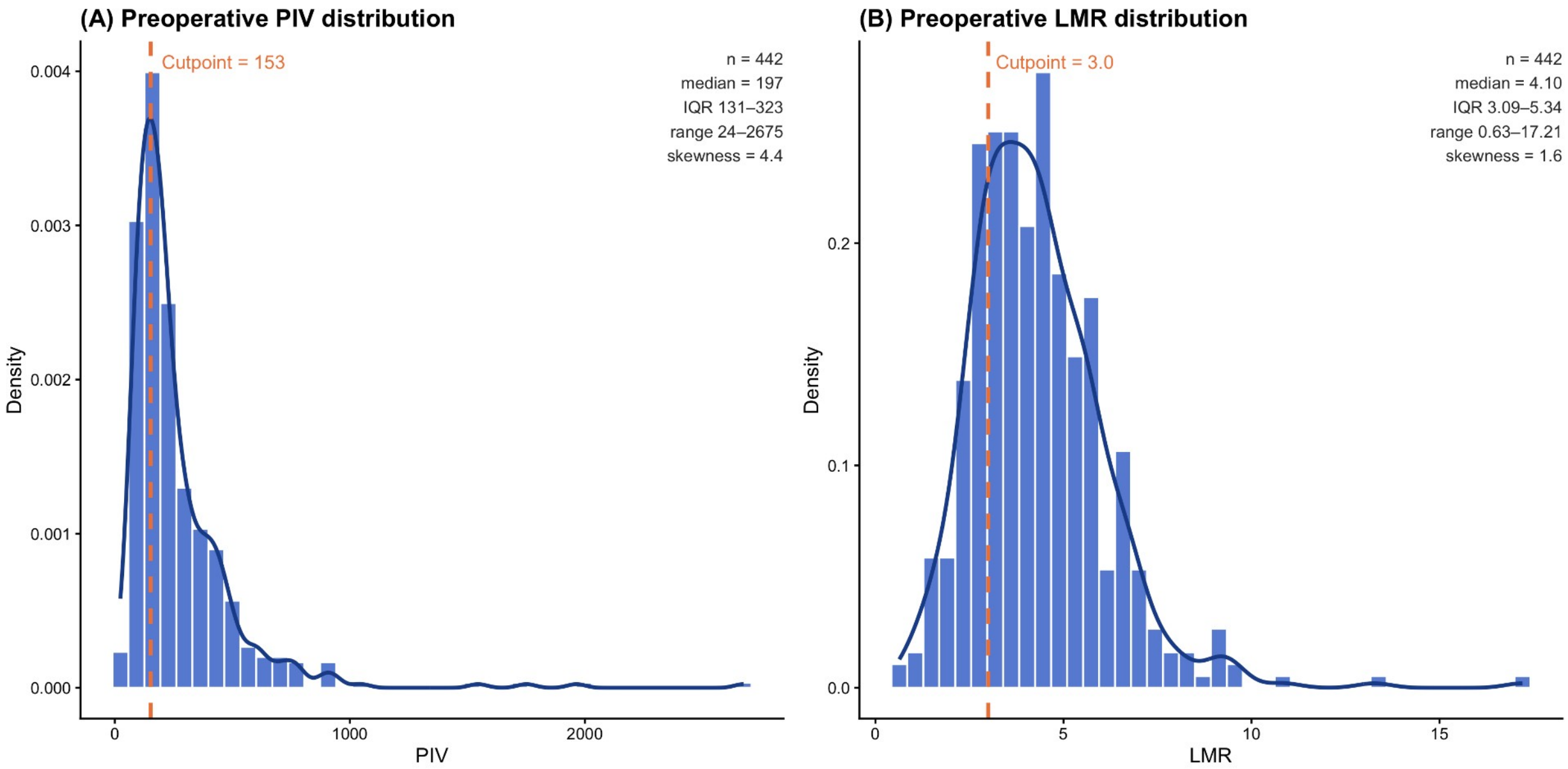

Supplementary Figure S2. Univariable (unadjusted) restricted cubic spline analysis of the association between preoperative pan-immune-inflammation value (PIV) and subsequent androgen deprivation therapy (ADT). PIV was log-transformed and truncated at the 95th percentile and modeled with three knots placed at the 10th, 50th and 90th percentiles. The shaded area represents the 95% confidence interval, the horizontal dashed line a hazard ratio of 1.0, and the vertical dashed line the Youden-derived cutpoint (PIV = 153). The association was significant overall (p = 0.017) without evidence of non-linearity (p = 0.169).

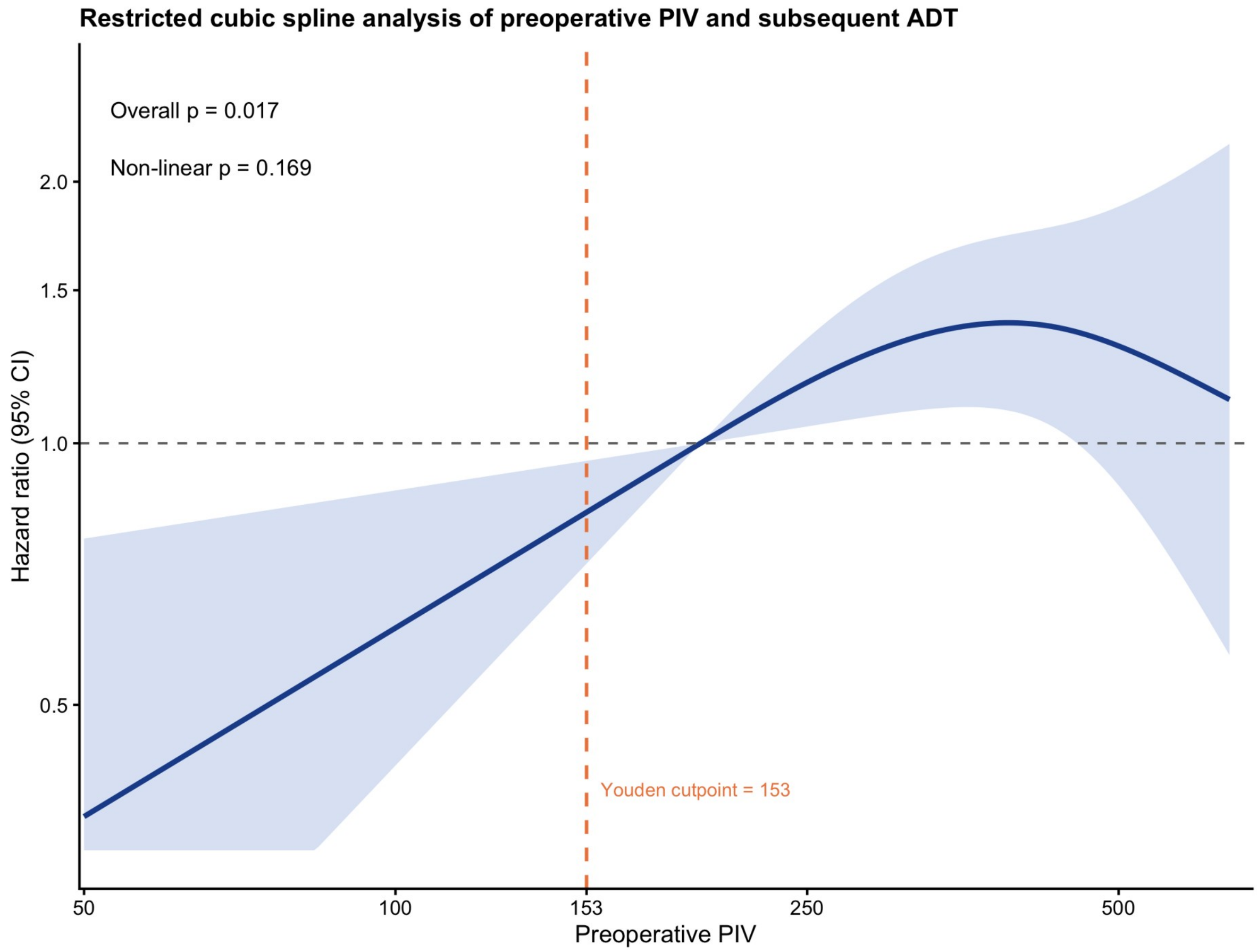

Supplementary Figure S3. Sensitivity analysis using tertiles of preoperative pan-immune-inflammation value (PIV). Patients were stratified into tertiles according to preoperative PIV (T1 ≤152, n = 148; T2 153-264, n = 147; T3 >264, n = 147). Kaplan-Meier curves are shown for (A) subsequent androgen deprivation therapy (ADT), (B) metastasis-free survival (MFS), and (C) overall survival (OS). Higher PIV tertiles were associated with poorer outcomes across all endpoints. Univariable Cox linear-trend p values were 0.005 for ADT, 0.003 for MFS, and < 0.001 for OS; the values printed within the Kaplan-Meier panels are overall log-rank p values (0.005, 0.009, and 0.001, respectively).

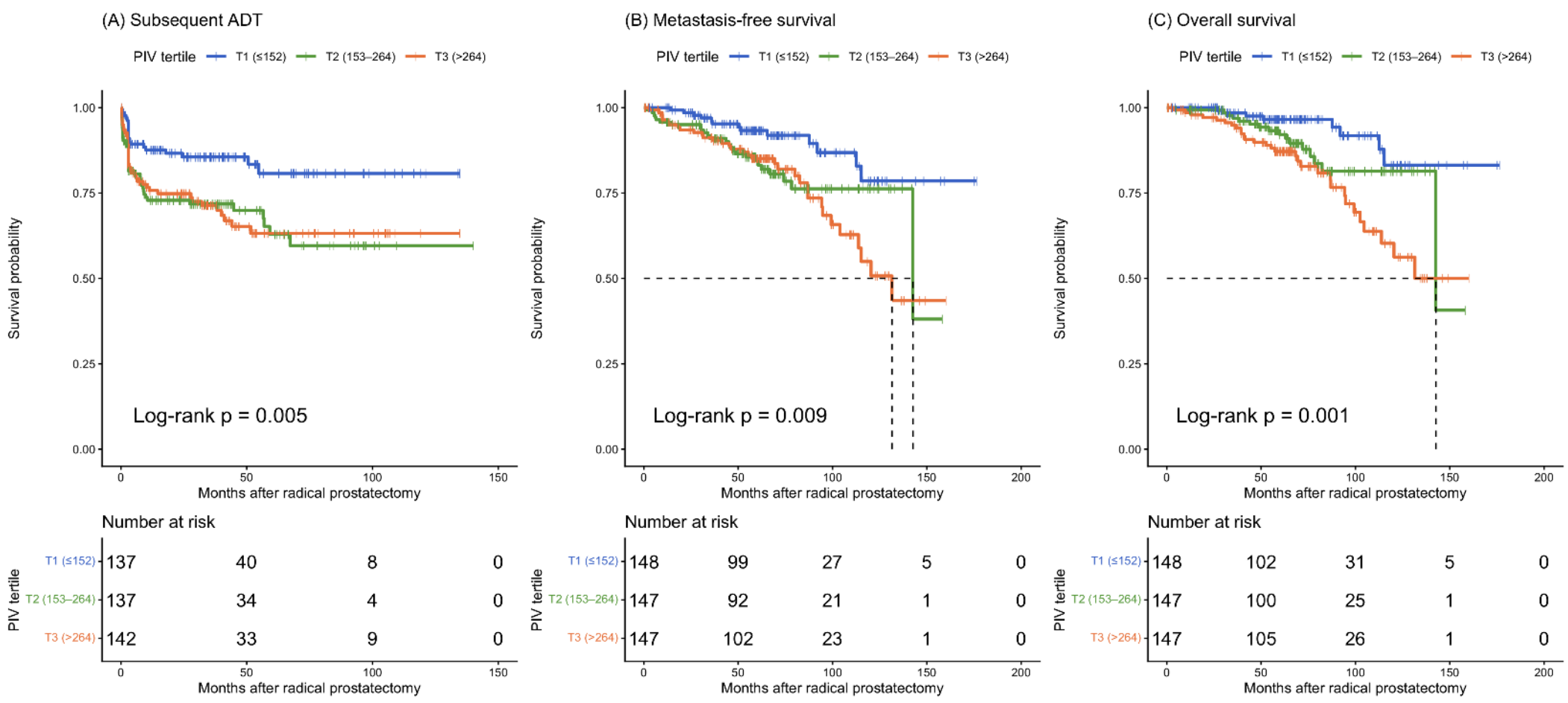

Supplement: Supplementary file 1 [file ijmsv23p3220s1.pdf]
